# Kinematic and Kinetic Adaptations to Step Cadence Modulation During Walking in Healthy Adults

**DOI:** 10.3390/jfmk11010053

**Published:** 2026-01-26

**Authors:** Joan Lluch Fruns, Maria Cristina Manzanares-Céspedes, Laura Pérez-Palma, Carles Vergés Salas

**Affiliations:** 1 Department of Clinic Sciences, University of Barcelona, 08028 Barcelona, Catalonia, Spain; joanlluch@ub.edu (J.L.F.); cverges@ub.edu (C.V.S.); 2 Department of Human Anatomy and Embryology, University of Barcelona, 08907 L’Hospitalet de Llobregat, Catalonia, Spain; mcmanzanares@ub.edu

**Keywords:** step cadence, human gait, biomechanics, plantar pressure, motor control, rehabilitation

## Abstract

**Background:** Walking cadence is commonly adjusted in sport and rehabilitation, yet its effects on spatiotemporal gait parameters and regional plantar pressure distribution under controlled speed conditions remain incompletely characterized. Therefore, this study aimed to determine whether imposed cadence increases at a constant walking speed would (i) systematically reduce temporal gait parameters while preserving inter-limb symmetry and (ii) be associated with region-specific increases in forefoot plantar loading, representing the primary novel contribution of this work. **Methods:** Fifty-two adults walked at three imposed cadences (110, 120, 130 steps·min^−1^) while maintaining a fixed treadmill speed of 1.39 m·s^−1^ via auditory biofeedback. Spatiotemporal parameters were recorded with an OptoGait system, and plantar pressure distribution was measured using in-shoe pressure insoles. Normally distributed variables were analyzed using repeated-measures ANOVA, whereas plantar pressure metrics were assessed using the Friedman test, followed by Wilcoxon signed-rank post-hoc comparisons with false discovery rate (FDR) correction. Associations between temporal parameters and plantar loading metrics (peak pressure, pressure–time integral) were examined using Spearman’s rank correlation with FDR correction (α = 0.05). **Results:** Increasing cadence produced progressive reductions in gait cycle duration (~8–10%), contact time (~7–8%), and step time (all *p* < 0.01), while inter-limb symmetry indices remained below 2% across conditions. Peak plantar pressure increased significantly in several forefoot regions with increasing cadence (all p_FDR < 0.05), whereas changes in the first ray were less consistent across conditions. Regional forefoot pressure–time integral also increased modestly with higher cadence (p_FDR < 0.01). Spearman’s correlations revealed moderate negative associations between temporal gait parameters and global plantar loading metrics (ρ = −0.38 to −0.46, all p_FDR < 0.05). **Conclusions:** At a constant walking speed, increasing cadence systematically shortens temporal gait components and is associated with small but consistent region-specific increases in forefoot plantar loading. These findings highlight cadence as a key temporal constraint shaping plantar loading patterns during steady-state walking and support the existence of concurrent temporal–mechanical adaptations.

## 1. Introduction

Human walking is a highly coordinated motor process that integrates neuromuscular control, joint dynamics, and the regulation of ground reaction forces to ensure a stable and efficient locomotor pattern [1,2]. Among the variables that modulate this pattern, step cadence—defined as the number of steps taken per minute—is one of the most sensitive and clinically relevant [3]. Cadence directly influences gait cycle timing, load distribution, and the overall mechanical efficiency of movement [1,2,3,4].

Stride-to-stride variability is considered a key indicator of neuromotor control and movement consistency [1,5]. In walking, a minimum level of steadiness is required to allow intentional stride modulations (e.g., obstacle avoidance or adaptive gait adjustments) [5]. In this context, cadence represents a major temporal constraint shaping locomotor control [3].

A considerable body of research has examined how modifying cadence affects running mechanics, showing that controlled increases in step frequency can reduce joint loading, enhance dynamic stability, and improve energetic efficiency [6,7,8]. However, the specific effects of cadence modulation during walking at a constant velocity remain comparatively underexplored, despite their relevance for postural control, rehabilitation, and functional gait assessment [1,3,4,9,10].

Most previous studies have manipulated stride length and stride frequency simultaneously, making it difficult to isolate their independent mechanical effects [11]. Addressing this limitation, Mercer et al. [11] independently manipulated stride length and stride frequency and demonstrated that these variables exert distinct biomechanical influences. Their work supports the need to experimentally constrain cadence under constant speed conditions to isolate its specific effects [11].

Studies by Almonroeder et al. [8], Heiderscheit et al. [12], Wellenkotter et al. [6], and Chumanov et al. [7] demonstrated that cadence can alter muscle recruitment patterns and the temporal components of stance (contact time and the corresponding stance proportion) independently of step length, suggesting a direct influence on neuromechanical function of the lower limbs [6,7,8,12]. In parallel, high-resolution kinematic systems such as OptoGait^®^ have enabled precise characterization of spatiotemporal adjustments and their association with locomotor stability [13].

From a stability perspective, Hak et al. [14] showed that increasing stride frequency enhances medio-lateral margins of stability during walking, independently of stride length and walking speed [14]. These findings highlight cadence as a modifiable control parameter for frontal-plane stability and balance regulation [14].

Consistent with this methodological approach, Mercer et al. [11] successfully used external auditory cues (metronome) to experimentally constrain stride frequency under constant speed conditions, providing further support for the experimental design adopted in the present study [11].

Using external auditory cues and visual feedback, Danion et al. [5] experimentally constrained stride frequency and reported a U-shaped relationship between cadence and gait variability, with minimal variability observed around ~1 Hz (≈120 steps·min^−1^), suggesting the existence of an optimal cadence for motor control. This finding further supports the relevance of cadence manipulation as an experimental and clinical tool [5].

Previous studies using rhythmic auditory cueing (RAC) have further demonstrated its effectiveness in systematically modulating spatiotemporal gait parameters. Yu et al. [15] showed that walking at 110% of an individual’s preferred cadence significantly increased stride length, cadence, and gait speed in healthy young adults [15]. Similarly, Minino et al. [10] reported that RAC frequencies equal to or slightly higher than natural cadence (100–110%) reduced gait cycle duration and trunk sway, suggesting improved gait stability [10]. Together, these findings support the use of personalized metronomic cueing as an effective strategy to modulate gait timing and dynamic stability.

From a kinetic perspective, plantar instrumentation systems such as F-Scan^®^ have shown that increasing cadence modifies the distribution of plantar pressures, particularly within the forefoot and toe regions [16]. These changes have been described in previous work as consistent with variations in regional plantar loading during late stance, although the underlying mechanical processes remain unclear [4,9,16].

Recent investigations by Lung et al. [4], Zhu et al. [16], and Wellenkotter et al. [6] suggested that higher cadence may improve plantar pressure distribution and stability [4,6,16], whereas Wang et al. [3], Heiderscheit et al. [12], and Almonroeder et al. [8] associated cadence modulation with reduced risk of overuse injuries [3,8,12]. Nonetheless, most prior studies examined kinematic and kinetic parameters separately, without integrating them within a unified experimental framework. This limits our understanding of how temporal gait adaptations influence mechanical loading and propulsive function simultaneously.

Consequently, a knowledge gap remains regarding how step cadence modulation jointly affects spatiotemporal and plantar kinetic behavior during walking at a constant velocity [3,4,11,16]. Clarifying this relationship is essential for understanding motor-control strategies and optimizing their potential applications in both clinical and performance contexts [1,5,10,14].

Therefore, the aim of the present study was to examine the effects of controlled cadence increases on spatiotemporal gait parameters and plantar pressure distribution during walking at a constant velocity in healthy adults [3,4,11,16].

While cadence-related reductions in temporal gait parameters have been previously reported and are therefore considered confirmatory, the primary novel contribution of this study is the quantification of how cadence-driven temporal constraints relate to region-specific forefoot plantar loading [3,10,16].

Accordingly, we hypothesized that increasing walking cadence would

(i) systematically reduce temporal gait parameters, including step time, contact time, and gait cycle duration, while preserving inter-limb symmetry, and
(ii) be associated with modest increases in regional forefoot plantar loading, as reflected by peak plantar pressure and pressure–time integral (PTI), indicating a coordinated temporal–mechanical adjustment.

## 2. Materials and Methods

### 2.1. Participants

Fifty-two healthy adults (26 males, 26 females; age 25.3 ± 4.2 years; height 1.73 ± 0.09 m; body mass 68.4 ± 8.5 kg) participated in this study. No participant reported musculoskeletal, neurological, or lower-limb pathology within the previous six months. All were autonomous walkers with a body mass index within the normal range (18.5–24.9 kg·m^−2^).

Sample size was determined based on previous within-subject studies on cadence modulation and plantar pressure variables, which have used similar or smaller samples. Because each participant served as their own control, this repeated-measures design reduced inter-individual variability and increased statistical power. In addition, the need to obtain stable estimates across multiple plantar regions was considered. For these reasons, 52 participants were recruited, allowing robust comparisons across the three experimental conditions and accounting for potential exclusions due to sensor artifacts.

This study adhered to the Declaration of Helsinki (2013 revision) and received approval from the Ethics Committee of the University of Barcelona (UB-2014-23). Written informed consent was obtained from all participants.

### 2.2. Experimental Design

A within-subject, repeated-measures design was employed. Each participant completed three cadence-controlled walking conditions: 110 steps·min^−1^ (baseline), 120 steps·min^−1^ (intermediate), and 130 steps·min^−1^ (high cadence).

Treadmill speed was fixed at 1.39 m·s^−1^ to ensure that observed gait adaptations were attributable solely to cadence modulation. The order of the three conditions was assigned using a counterbalanced Latin-square design to minimize potential order effects.

**Figure 1:**
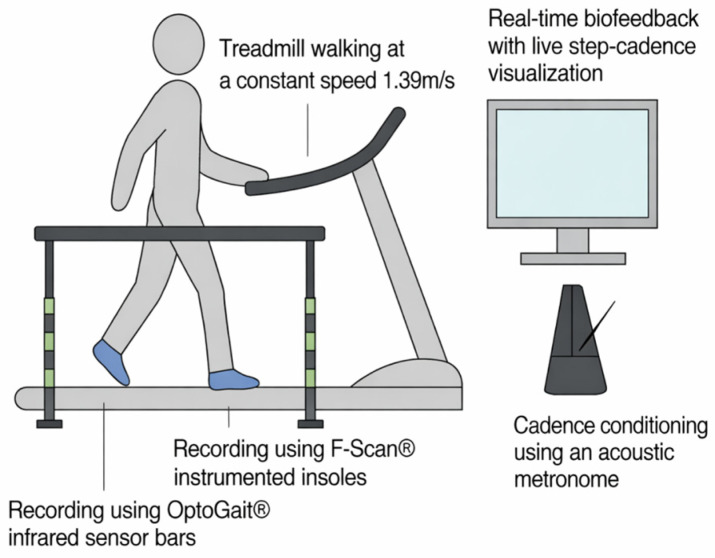
Experimental setup.

Participants walked on a motorized treadmill (NordicTrack^®^ T12, iFIT Health & Fitness Inc., Logan, UT, USA), while cadence was externally imposed using an auditory metronome (Korg MA-2, Korg Inc., Tokyo, Japan), with each acoustic signal corresponding to a foot contact. Cadence compliance was continuously monitored through a combination of the metronome and real-time visual biofeedback provided by the OptoGait system. Step frequency (±1 step·min^−1^) was displayed on a front-mounted monitor (Figure 1), allowing both participants and investigators to verify maintenance of the target cadence throughout each trial.

Participants walked on a motorized treadmill at a fixed speed of 1.39 m·s^−1^ while cadence was externally controlled using an auditory metronome and visual feedback display. Spatiotemporal data were acquired using OptoGait^®^ infrared bars and plantar pressure data were acquired using Tekscan F-Scan^®^ in-shoe sensors. All data streams were synchronized and recorded continuously.

### 2.3. Measurement Instruments

#### 2.3.1. Kinematic Analysis

Spatiotemporal data were processed using OptoGait Analysis Suite v1.12 (Microgate Srl., Bolzano, Italy) consisting of parallel transmitting and receiving infrared bars operating at a sampling frequency of 1000 Hz with a spatial resolution of 1 cm. Initial contact and toe-off events were automatically detected using the manufacturer’s standard algorithm.

The following variables were extracted: gait cycle duration (s), cadence (steps·min^−1^), step time (s), stride length (cm), step length (cm), contact phase (%), and swing phase (%).

Right and left limbs were initially processed separately to examine potential inter-limb differences. Descriptive analyses revealed minimal and consistent differences between limbs across all cadence conditions, with step-length differences <1 cm, step-time differences ≤0.01 s, and contact-phase differences <0.5 percentage points. This pattern indicated a high degree of functional symmetry during treadmill walking.

Because the present study was not statistically powered to detect subtle limb-specific effects—particularly for plantar pressure variables—and the main objective was to characterize global cadence-related adaptations, left and right values were averaged to obtain limb-averaged measures for all subsequent analyses.

For each cadence condition, spatiotemporal variables were averaged over the steady-state walking period.

#### 2.3.2. Kinetic Analysis

Plantar pressure data were processed using Tekscan F-Scan Research 8.0 (Tekscan Inc., South Boston, MA, USA)equipped with resistive sensor insoles. Data were recorded at a sampling frequency of 100 Hz, according to manufacturer specifications, and calibrated for each participant according to body mass following the manufacturer’s recommended procedure.

Participants wore their own athletic footwear fitted with F-Scan^®^ insoles. OptoGait and F-Scan recordings were initiated simultaneously at trial onset and later aligned offline using the same retained steady-state steps, ensuring temporal synchronization between spatiotemporal and plantar pressure data. According to manufacturer specifications, sensor accuracy was approximately ±5% of full-scale load.

The plantar surface was divided into five anatomical regions: hallux, lesser toes, first ray, central rays, and fifth ray. For each region, peak plantar pressure (kPa) was defined as the maximum pressure value observed during stance, and pressure–time integral (PTI, kPa·s) was calculated as the integral of pressure over stance time. Left and right foot data were averaged to obtain limb-averaged values for each condition.

Because step-level plantar pressure variables typically exhibit skewed distributions and occasional extreme values, regional peak pressure and PTI were summarized using step-level medians for each participant and condition. These subject-level median values were then used for all subsequent statistical and correlational analyses.

Left and right foot data were initially processed separately. Given the absence of relevant inter-limb differences and the primary objective of characterizing global cadence-related adaptations, limb-averaged values were subsequently computed for each condition.

### 2.4. Experimental Procedure

Participants first completed a 2 min familiarization period on the treadmill to practice synchronizing their steps with the metronome and to ensure a comfortable gait at the imposed belt speed. Footwear was then fitted with the F-Scan insoles, and sensors were zeroed in an unloaded seated position according to the manufacturer’s recommendations.

Each cadence condition (110, 120, 130 steps·min^−1^) lasted 90 s. Participants were instructed to match every step to the auditory cue of the metronome. Real-time cadence feedback displayed on the treadmill monitor was used by both participants and investigators to verify attainment and maintenance of the target cadence. In addition, the OptoGait software provided a cadence variability indicator, which was monitored to confirm stepping pattern stability during each condition.

The initial transition phase and the final deceleration phase of each trial were later excluded from analysis (see Section 2.5). For subsequent analyses, only periods in which cadence remained within ±1 step·min^−1^ of the target value were retained. The central steady-state portion of each recording was used for spatiotemporal and plantar pressure processing.

OptoGait and F-Scan data were recorded simultaneously and exported for offline alignment and analysis, as described in Section 2.5.

### 2.5. Data Processing

For each participant and cadence condition, raw OptoGait and F-Scan recordings were visually inspected to identify the onset of stable walking. No automated algorithm was used to define steady state. Instead, steady-state gait was identified based on real-time cadence stabilization verified via the OptoGait biofeedback displayed on the treadmill monitor.

Specifically, the steady-state phase was considered to begin once participants consistently maintained the target cadence within ±1 step·min^−1^. The initial steps preceding attainment of the target cadence were excluded. Analogously, the final steps of each recording were removed to avoid deceleration artifacts.

All analyses were therefore based on gait cycles occurring within the central 60 s steady-state interval (typically 55–65 cycles per trial), during which cadence stability was confirmed.

#### 2.5.1. Spatiotemporal Variables (OptoGait)

Foot-contact and toe-off events were detected using the manufacturer’s standard LED-interruption algorithm. Valid gait cycles were defined as those with complete and artifact-free detection of initial contact and toe-off events.

For each valid gait cycle, step time, contact time, gait cycle duration, and stance phase percentage were computed. Values were averaged across all valid cycles within the steady-state segment. Cycles showing incomplete foot contact, double strikes, cadence irregularities, or signal-detection artifacts were excluded.

Right and left limbs were processed independently for symmetry analysis. Because all analyses were restricted to the same central 60 s steady-state interval for all participants, the number of retained gait cycles was comparable across participants.

#### 2.5.2. Plantar Pressure Variables (F-Scan)

**Figure 2:**
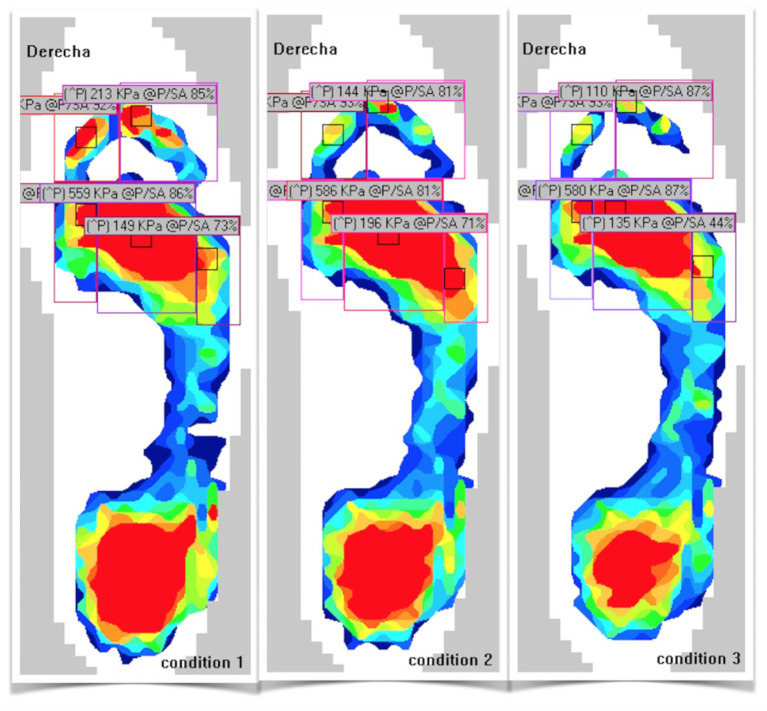
Schematic division of the plantar surface into anatomical regions.

Plantar pressure data were processed using the same steady-state steps retained for the spatiotemporal analysis. The plantar surface was divided into five anatomical regions—hallux, lesser toes, first ray, central rays, and fifth ray—as illustrated in Figure 2.

For each predefined foot region, two outcome metrics were computed: peak plantar pressure (kPa) and pressure–time integral (PTI; kPa·s), obtained by numerical integration over stance.

Both variables were summarized as the median [Q1; Q3] across all valid steps of the steady-state interval. Steps affected by sensor dropout, edge-loading artifacts, or non-physiological values were excluded. Right and left limbs were processed independently prior to statistical analysis.

Spatiotemporal data were processed using OptoGait Analysis Suite v1.12 and plantar pressure data were processed using Tekscan F-Scan Research 8.0.

A schematic division of the plantar surface into anatomical regions was used for plantar pressure analysis. The forefoot was segmented into five regions: hallux, lesser toes, first ray, central rays, and fifth ray. This standardized regional mask was applied to all trials to extract regional peak plantar pressure and pressure–time integral (PTI) values.

### 2.6. Statistical Analysis

All statistical analyses were performed using Python (version 3.12.7; Python Software Foundation, Wilmington, DE, USA). Statistical significance was set at *p* < 0.05. Distributional characteristics of the data were inspected visually (histograms and Q–Q plots). Spatiotemporal gait parameters are reported as the mean ± standard deviation, whereas plantar pressure variables are reported as the median with interquartile range [Q1–Q3].

Spatiotemporal gait parameters (stride length, step length, step time, contact time, gait cycle duration, contact phase, and swing phase) were analyzed using one-way repeated-measures analysis of variance (RM-ANOVA), with cadence condition (110, 120, and 130 steps·min^−1^) as the within-subject factor. Cadence was experimentally imposed and therefore not treated as a dependent variable; instead, it was reported descriptively as a manipulation check. When significant main effects were detected, post-hoc pairwise comparisons were performed using Bonferroni-adjusted comparisons. In addition to *p*-values, partial eta squared (ηp^2^) was calculated and reported as a measure of effect size. Effect sizes were interpreted according to conventional thresholds: for ηp^2^, 0.01 (small), 0.06 (moderate), and ≥0.14 (large).

Plantar pressure outcomes, including peak plantar pressure (PPP, kPa) and pressure–time integral (PTI, kPa·s), were analyzed using non-parametric methods due to skewed distributions and the presence of extreme values. All analyses were performed on limb-averaged values obtained from n = 52 participants. Peak plantar pressure (PPP) was defined as the maximum pressure value recorded within each plantar region across the entire contact phase of the gait cycle (0–100%). PPP values were extracted from time-normalized pressure data sampled at successive instants of stance (0%, 10%, 20%, …, 100%) for both right and left feet.

Differences across cadence conditions were assessed using the Friedman test for repeated measures, followed by Wilcoxon signed-rank tests for pairwise comparisons. Kendall’s W was reported as an effect size for Friedman tests and Wilcoxon’s r was calculated for pairwise comparisons. For both Kendall’s W and Wilcoxon’s r, effect sizes were interpreted as small (0.10), moderate (0.30), and large (≥0.50). To control for multiple comparisons across plantar regions within each plantar pressure metric (peak pressure and PTI), *p*-values were adjusted using the Benjamini–Hochberg false discovery rate (FDR) procedure, applied separately within each outcome family (peak pressure and PTI).

Associations between temporal spatiotemporal gait parameters (contact time and gait cycle duration) and plantar loading outcomes (forefoot peak plantar pressure and pressure–time integral) were explored using Spearman’s rank correlation coefficients (ρ). For each participant and variable, values were first averaged within each cadence condition (110, 120, and 130 steps·min^−1^) and subsequently averaged across the three conditions to obtain a single subject-level value for correlation analyses. This approach ensured that each participant contributed one independent observation, thereby avoiding pooling of repeated measures and minimizing within-subject dependence. Exploratory correlations involving contact phase (%) were also computed to facilitate comparison with previous gait studies. For each set of correlations, *p*-values were adjusted for multiple testing using FDR correction within each correlation family.

### 2.7. Availability of Materials, Data, Code, and Protocols

The data presented in this study are not publicly available due to ethical and privacy considerations but are available from the corresponding author upon reasonable request. Data processing, statistical analyses, and figure generation were performed using custom scripts developed in Python (version 3.12.7). The analysis workflow and code can be shared upon reasonable request.

### 2.8. Generative AI Use Statement

Generative artificial intelligence (GenAI) tools were not used to generate, analyze, interpret, or create any scientific content, data, figures, or methodological procedures in this study. GenAI was used exclusively for superficial text editing (grammar, spelling, punctuation, and formatting) using ChatGPT (GPT-4, OpenAI, San Francisco, CA, USA), in accordance with MDPI guidelines.

## 3. Results

Cadence manipulation from 110 to 130 steps·min^−1^ produced systematic changes in spatiotemporal gait parameters and regional plantar pressure outcomes. Descriptive spatiotemporal results are summarized in Table 1, plantar pressure outcomes in Table 2, and correlation analyses in Table 3.

### 3.1. Spatiotemporal Parameters

Participants successfully maintained the imposed treadmill speed of 1.39 m·s^−1^ across all cadence conditions. Descriptive limb-specific analyses revealed minimal and consistent differences between right and left limbs across all conditions. Step length differences remained below 1 cm (≤1.4%), step time differences were ≤0.01 s, and contact phase differences were <0.5 percentage points. This pattern indicates a high degree of functional symmetry during treadmill walking.

Accordingly, inter-limb asymmetry values for step time and other temporal variables remained below 2.2%, with no systematic differences between conditions. For this reason, and in line with the primary objective of characterizing global cadence-related adaptations, spatiotemporal outcomes are presented as limb-averaged values.

Cadence was externally imposed and is therefore reported descriptively as a manipulation check rather than analyzed as an outcome variable. As expected, cadence differed significantly across conditions, confirming compliance with the experimental protocol (Table 1).

**Figure 3:**
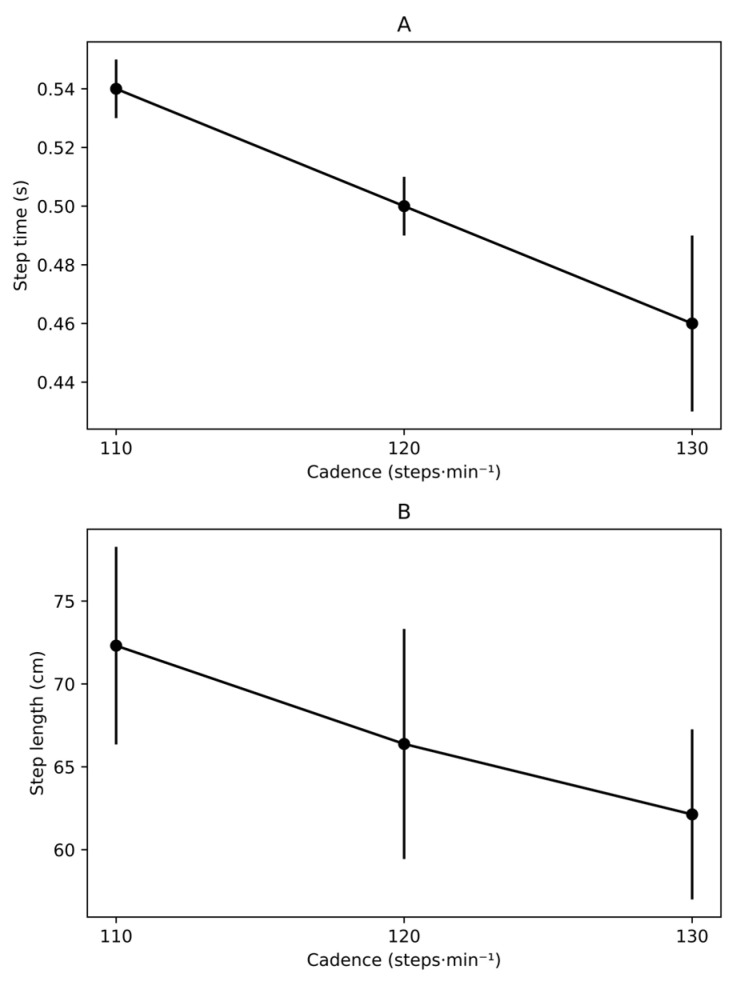
Spatiotemporal adaptations to cadence modulation during treadmill walking. (**A**) Step time (s) and (**B**) step length (cm) across cadence conditions (110, 120, and 130 steps·min^−1^) at a constant treadmill speed (1.39 m·s^−1^). Values are presented as mean ± standard deviation. Inferential statistics are provided in Table 1.

As expected, temporal gait parameters varied systematically across cadence conditions (Table 1). Figure 3 provides a graphical visualization of these trends across conditions to facilitate interpretation. Step time decreased monotonically across conditions (110 → 120 → 130 steps·min^−1^), from approximately 0.54 s at 110 steps·min^−1^ to 0.46 s at 130 steps·min^−1^ (*p* < 0.001). Gait cycle duration showed a similar pattern, with an overall reduction of approximately 8–10% across the cadence range (*p* < 0.001).

Consistent with the constant treadmill speed, spatial parameters also adapted to cadence modulation. Stride length decreased from 144.63 ± 11.93 cm at 110 steps·min^−1^ to 124.23 ± 10.25 cm at 130 steps·min^−1^ (*p* < 0.001), with a corresponding reduction in step length from 72.31 ± 5.96 cm to 62.13 ± 5.13 cm (*p* < 0.001).

Stance phase percentage exhibited a modest increase with higher cadence, rising from 67.80 ± 3.36% at 110 steps·min^−1^ to 69.67 ± 3.63% at 130 steps·min^−1^ (*p* = 0.002), accompanied by a complementary reduction in swing-phase percentage (*p* < 0.001). Contact time, derived as the product of gait cycle duration and stance phase percentage, decreased progressively across conditions, reflecting the shortening of both gait cycle duration and step time with increasing cadence.

Descriptive values (mean ± standard deviation) and statistical outcomes for all spatiotemporal variables are reported in Table 1. Variables were not normalized to anthropometric characteristics (e.g., body mass or leg length) because the analyses focused on within-subject comparisons across cadence conditions.

### 3.2. Regional Plantar Pressure Distribution

Because plantar pressure variables exhibited skewed distributions and the presence of extreme values, data are reported as medians [Q1–Q3] (Table 2). Friedman tests revealed significant cadence effects in four of the five forefoot regions. From 110 to 130 steps·min^−1^, peak plantar pressure increased significantly in the hallux, lesser toes, central rays, and fifth ray (all p_FDR < 0.05), whereas no significant change was observed in the first-ray region (p_FDR > 0.05).

**Figure 4:**
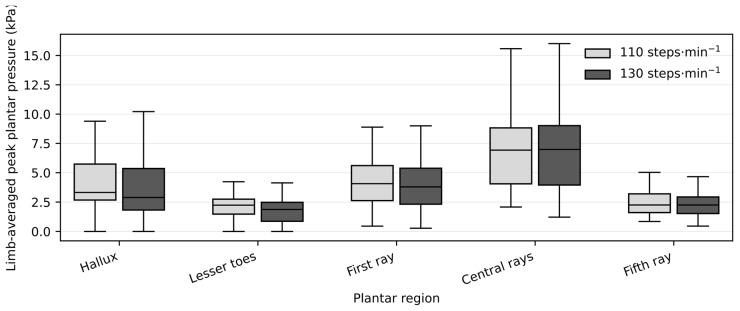
Regional peak plantar pressure at low and high cadence conditions.

Across these regions, relative increases in peak plantar pressure with higher cadence were generally modest, on the order of approximately 8–10% in the lesser toes and fifth ray, with smaller but still significant increases in the hallux and central rays. These regional changes are illustrated in Figure 4. Exact medians, interquartile ranges, Friedman test statistics, and Wilcoxon effect sizes (r) for peak pressures are provided in Table 2.

**Figure 5:**
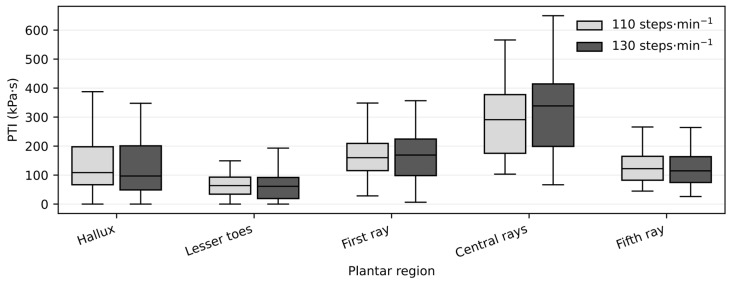
Pressure–time integral (PTI) at low and high cadence conditions.

Forefoot pressure–time integral (PTI) also increased modestly with cadence (p_FDR < 0.01), indicating a small but consistent elevation in cumulative plantar loading when walking at higher step rates (Figure 5). Regional PTI values showed similar cadence-related trends, with statistically significant increases in selected forefoot regions after FDR correction.

Box plots show median values and interquartile ranges for limb-averaged peak plantar pressure (kPa) across plantar regions at 110 and 130 steps·min^−1^. Statistical comparisons between cadence conditions were performed using Wilcoxon signed-rank tests, with false discovery rate (FDR) correction applied across plantar regions. Significant differences after FDR correction are indicated (p_FDR < 0.05).

Box plots show median values and interquartile ranges for limb-averaged PTI (kPa·s) across plantar regions at 110 and 130 steps·min^−1^. Statistical comparisons between cadence conditions were performed using Wilcoxon signed-rank tests, with false discovery rate (FDR) correction applied across plantar regions. Significant differences after FDR correction are indicated (p_FDR < 0.05).

### 3.3. Associations Between Spatiotemporal and Plantar Pressure Variables

Spearman’s rank correlations were used to examine whether temporal gait parameters were associated with F plantar loading metrics (Table 3). After FDR adjustment, longer contact time was moderately associated with lower forefoot peak plantar pressure (ρ = −0.46, p_FDR = 0.001). Stance phase percentage also showed a moderate negative association with forefoot peak plantar pressure (ρ = −0.41, p_FDR = 0.006). In addition, longer gait cycle duration was associated with lower forefoot PTI (ρ = −0.38, p_FDR = 0.011). No other correlations between temporal parameters and forefoot plantar loading metrics remained significant after FDR correction.

Exploratory correlations between spatiotemporal parameters and regional plantar pressure metrics (peak pressure and PTI in the hallux, lesser toes, first ray, central rays, and fifth ray) yielded small effect sizes (|ρ| generally < 0.30), and none survived FDR correction (all p_FDR > 0.05). These regional correlation results are summarized in Supplementary Table S1.

### 3.4. Summary of Main Quantitative Findings

- Increasing cadence from 110 to 130 steps·min^−1^ at constant walking speed led to shorter step time, contact time, and gait cycle duration, with small increases in stance-phase and pre-swing percentages and stable inter-limb symmetry.
- Peak plantar pressure increased significantly in the hallux, lesser toes, central rays, and fifth ray, whereas the first-ray region remained unchanged. Regional PTI showed modest, region-specific increases with cadence.
- Moderate negative Spearman correlations were found between key temporal parameters (contact time, stance-phase percentage, gait cycle duration) and global plantar pressure metrics (peak plantar pressure and pressure–time integral).

## 4. Discussion

The present study investigated how cadence modulation at a constant walking speed influences the temporal and spatial organization of gait and its relationship with plantar loading. The primary finding was that increasing cadence induced a marked temporal compression of stance, as evidenced by progressive reductions in contact time and gait cycle duration, alongside a concomitant decrease in stride length. Despite this reduction in absolute contact time, the relative contribution of the contact phase to the gait cycle increased, indicating a reorganization of temporal proportions rather than a simple shortening of stance. These results highlight contact time as a central temporal variable associated with cadence-related gait adaptations and provide a coherent temporal framework for interpreting the observed changes in plantar loading.

Our results are consistent with previous studies employing rhythmic auditory cueing. Yu et al. [15] reported that a 110% RAC condition significantly increased cadence and stride length in healthy young adults, supporting the effectiveness of metronomic stimulation for gait modulation. Similarly, Minino et al. [10] showed that RAC frequencies slightly above natural cadence improved temporal gait parameters and reduced trunk sway, suggesting enhanced dynamic stability. Together, these findings reinforce the validity of using metrorhythmic cues to experimentally and clinically manipulate gait timing.

### 4.1. Kinematic Adaptations

Increasing cadence produced clear and systematic reductions in gait cycle duration, step time, and contact time, together with a modest increase in the stance proportion of the gait cycle. This pattern indicates that participants completed the stance more rapidly in absolute terms (shorter contact time), while the stance share of the gait cycle increased slightly. These findings align with previous evidence indicating that cadence acts as a primary timing-regulation mechanism influencing postural control, stability, and intersegmental coordination [1,2,5,6,9].

The consistently low step time asymmetry (<2%) across all cadence conditions indicates that these adjustments occurred without detectable left–right temporal imbalances [13]. This supports the notion that cadence increments are implemented through system-wide neuromuscular timing adaptations rather than limb-specific corrective actions [1,10], although the present design cannot directly confirm the underlying control mechanisms.

In this context, Danion et al. [5] reported a U-shaped relationship between stride frequency and gait variability, with minimal variability observed around ~1 Hz (≈120 steps·min^−1^). They proposed that this optimal cadence reflects the natural resonance frequency of the locomotor system, at which neuromuscular involvement and internal perturbations are minimized [5]. Our findings are compatible with this framework, as temporal adaptations occurred without loss of symmetry or apparent destabilization, supporting cadence as a global control parameter [1,10].

Furthermore, Mercer et al. [11] demonstrated that stride length and stride frequency exert distinct biomechanical effects [11]. In the present study, cadence increments were accompanied by systematic reductions in stride length, suggesting that part of the observed kinematic reorganization reflects combined temporal constraints and geometric adjustments of the lower limbs.

Consistent with these findings, Hak et al. [14] showed that increasing stride frequency enhances medio-lateral margins of stability during walking, independently of stride length and walking speed [14]. This supports the interpretation that cadence manipulation may contribute to improved frontal-plane stability without compromising dynamic consistency of gait.

### 4.2. Kinetic Adaptations

Higher cadence was associated with increased plantar pressures in distal and lateral forefoot regions, particularly the lesser toes and fifth ray, while pressures under the first ray remained relatively stable [4,16]. This distal–lateral redistribution is compatible with a center-of-pressure progression that shifts laterally and distally during terminal stance under faster temporal constraints [16]. However, this interpretation remains speculative because center-of-pressure trajectories and ground-reaction forces were not recorded.

Hak et al. [14] reported that increased cadence is associated with larger medio-lateral margins of stability, which may require greater engagement of lateral and distal forefoot structures to control frontal-plane balance [14]. This mechanism may partly explain the increased pressures observed in the lateral forefoot regions in the present study.

Additionally, Mercer et al. [11] showed that changes in stride length, rather than stride frequency per se, primarily influence impact-related mechanical responses [11]. Given that cadence increases in the present study were accompanied by shorter stride lengths, this mechanism may partly explain the observed redistribution of plantar pressures. Shorter steps may alter lower-limb geometry at contact, thereby modifying load transfer across the forefoot.

Although vertical ground-reaction-force data were not collected, the combination of reduced contact time and increased localized forefoot pressure suggests a greater temporal concentration of plantar loading [16]. Similar patterns have been reported in contexts involving increased activation of digital flexors and peroneal muscles, potentially contributing to mediolateral stability and late-stance propulsion [9]. Because electromyography was not included, these interpretations should be regarded as hypotheses informed by prior work rather than conclusions drawn directly from our data.

Overall, the present results are consistent with the concept that cadence increments compress stance-related events in time while coinciding with greater loading over distal propulsive structures [4,16]. However, the precise mechanical and neuromuscular mechanisms underlying this redistribution cannot be established from the present dataset.

### 4.3. Kinematic–Kinetic Integration

Correlation analyses revealed moderate associations between temporal gait parameters and forefoot plantar loading metrics, including forefoot peak plantar pressure and pressure–time integral (PTI). Within the cadence range examined, this pattern indicates that shorter stance-related time windows tended to co-occur with higher plantar loads. However, given the cross-sectional design and the moderate magnitude of the correlation coefficients, these findings should be interpreted as descriptive associations rather than causal relationships [4,16].

It should be noted that averaging values across cadence conditions emphasizes between-subject associations and may attenuate cadence-specific relationships.

This pattern parallels observations in fast walking, where reduced stance-related timeframes have been reported alongside greater forefoot loading without necessarily requiring increased total force production [4,16]. Mercer et al. [11] similarly emphasized that small modifications in gait pattern can substantially alter mechanical loading without major energetic penalties, supporting the functional relevance of cadence-driven adaptations [11].

The present findings therefore extend previous work by quantifying how cadence-related changes in temporal parameters relate to changes in regional plantar pressures under constant speed conditions [3,4,16]. Within this framework, the observed adjustments can be tentatively summarized as follows:

- Temporal: reduced step time and accelerated stance events [3,10];
- Mechanical: increased distal–lateral plantar loading [4,16];
- Functional: preservation of bilateral symmetry and locomotor stability proxies [13,14].

Importantly, these patterns do not imply specific motor-control strategies but rather describe concurrent adaptations observed under externally constrained cadence conditions. Therefore, any interpretation in terms of control mechanisms should be considered speculative.

These findings are compatible with the work of Hak et al. [14], who showed that cadence-related temporal adjustments can modify stability-related mechanical demands without affecting local dynamic stability. Together, this supports the notion that cadence-driven changes may selectively influence balance-related mechanical requirements rather than global locomotor stability. Nevertheless, direct neuromuscular and dynamic stability measures would be required to substantiate this interpretation.

### 4.4. Clinical Implications

These results parallel observations in fast walking, where reduced stance-related timeframes have been reported alongside increased forefoot loading without necessarily requiring higher total force production [4,16]. The present findings extend previous work by quantifying how cadence-related temporal adjustments relate to changes in regional plantar pressures under constant-speed conditions [3,4,16].

From a clinical perspective, Danion et al. [5] demonstrated that preferred gait patterns are not necessarily those associated with minimal variability, supporting the potential utility of cadence manipulation as a therapeutic strategy [5]. In this context, rhythmic auditory cueing (e.g., metronome-based interventions) has been shown to be an effective tool to externally modulate step frequency in both healthy and clinical populations [10,15]. Our results reinforce this concept, suggesting that externally imposed cadence could be explored as a tool to modulate gait timing and plantar loading patterns in rehabilitation settings, particularly in individuals with neurological or musculoskeletal disorders.

Together, these findings indicate that cadence-based interventions may have the potential to improve temporal organization and dynamic stability while simultaneously modulating plantar loading in clinical populations, such as individuals with stroke, Parkinson’s disease, or lower-limb osteoarthritis [1,14]. However, given the correlational nature of the present analysis and the absence of direct neuromuscular or stability measures, these interpretations should be considered preliminary and warrant confirmation in targeted clinical trials.

### 4.5. Limitations and Future Perspectives

The present findings may inform future work on cadence modulation as a tool to influence gait timing and plantar loading patterns. In particular, the observation that temporal reorganization occurred without measurable reductions in inter-limb symmetry [3,11,13] suggests that cadence manipulation could be explored as a strategy to adjust spatiotemporal parameters and regional forefoot loading in clinical populations. However, because the current data derive exclusively from young healthy adults, extrapolation to other populations should be approached with caution.

An additional methodological consideration relates to the externally imposed nature of cadence in the present protocol. Cadence was constrained using rhythmic auditory cueing, which may engage attentional and sensorimotor processes that differ from those involved in spontaneous self-selected walking. Consequently, the observed adaptations reflect responses to externally constrained temporal conditions rather than natural cadence regulation. This distinction should be considered when interpreting ecological validity and transferability to unconstrained overground walking.

Nevertheless, it is important to note that rhythmic auditory cueing delivered through simple devices such as headphones or smartphone applications represents a low-cost, accessible, and widely used strategy in both clinical practice and daily-life contexts. In this sense, the present experimental paradigm may reasonably approximate real-world scenarios in which individuals intentionally modulate step frequency using external rhythmic stimuli. Thus, while externally imposed cadence constitutes a methodological constraint, it also reflects a practically applicable intervention framework.

Several additional methodological limitations should be acknowledged. First, participants were allowed to wear their own athletic footwear, which may have influenced absolute plantar pressure magnitudes due to differences in midsole properties and shoe geometry [4,16]. Although our analyses focused on within-subject comparisons across cadence conditions—thereby minimizing inter-individual bias—this factor may still contribute to variability and should be controlled in future studies.

Second, foot morphology (e.g., arch structure, pes planus, or pes cavus) was not assessed. Given that foot posture is known to influence regional plantar pressure distribution, the absence of this information limits the generalizability of our findings and may partially explain inter-individual variability [2,9].

Third, gait variables were not normalized to anthropometric characteristics (e.g., body mass or leg length), which may affect absolute values and between-subject comparisons. Future research should consider normalization procedures to improve comparability across individuals [3,10].

In clinical populations with impaired motor control—such as peripheral neuropathy, stroke, or knee osteoarthritis—increasing cadence could potentially promote a more consistent temporal organization of gait, along with slightly higher stance proportions, with possible implications for joint or soft-tissue loading [3,10,14]. Similarly, in forefoot rehabilitation, moderate cadence increases might be explored experimentally to evaluate whether plantar loading can be redistributed toward distal segments and whether such changes relate to altered intrinsic foot muscle activation or dynamic stability [2,4,8,9,12,16]. These possibilities remain hypothesis-generating and would require targeted longitudinal studies incorporating standardized footwear, foot morphology assessment, and additional biomechanical measures (e.g., electromyography and ground reaction forces).

## 5. Conclusions

A controlled increase in walking cadence was associated with coordinated changes in temporal gait parameters and plantar loading patterns in healthy adults walking at a constant speed. Increasing cadence systematically shortened gait cycle duration, step time, and contact time (approximately 8–10%), while inter-limb temporal symmetry remained preserved across conditions.

Concurrently, peak plantar pressure increased modestly in several distal forefoot regions—including the lesser toes, fifth ray, hallux, and central rays—whereas first-ray loading remained relatively stable. Whole-foot pressure–time integral also showed a small but consistent increase at higher cadences, indicating a modest elevation in cumulative plantar loading.

Moderate negative associations were observed between key temporal parameters (contact time, stance phase percentage, and gait cycle duration) and whole-foot plantar loading metrics, suggesting that shorter stance-related time windows tend to co-occur with higher plantar loads. These relationships should be interpreted as descriptive, given the cross-sectional design and absence of direct neuromuscular or mechanical measurements.

Together, these findings characterize how cadence modulation reorganizes gait timing and plantar loading during steady-state walking without implying specific underlying control mechanisms. From a clinical perspective, cadence manipulation may warrant further investigation as a potential strategy for adjusting gait timing and plantar loading patterns in populations with motor impairments. However, extrapolation beyond healthy young adults requires caution, and future studies should incorporate longitudinal designs and additional biomechanical measures—including electromyography and ground reaction forces—to clarify the mechanisms and clinical relevance of cadence-related gait adaptations.

## Figures and Tables

**Table 1:** Limb-averaged spatiotemporal gait parameters across cadence conditions (mean ± SD) and effect sizes.

Variable	Con1 (110 Steps·min^−1^)	Con2 (120 Steps·min^−1^)	Con3 (130 Steps·min^−1^)	*p*-Value	ηp^2^
Cadence (steps·min^−1^)	110.51 ± 3.26	119.95 ± 2.04	129.55 ± 1.70	<0.001	0.946
Stride length (cm)	144.63 ± 11.93	134.02 ± 11.29	124.23 ± 10.25	<0.001	0.962
Step length (limb-avg) (cm)	72.31 ± 5.96	66.38 ± 6.94	62.13 ± 5.13	<0.001	0.790
Contact phase (limb-avg) (%)	67.80 ± 3.36	68.83 ± 4.43	69.67 ± 3.63	0.002	0.114
Swing phase (%)	32.15 ± 3.43	30.98 ± 3.74	30.33 ± 3.68	0.001	0.127
Step time (limb-avg) (s)	0.54 ± 0.01	0.50 ± 0.01	0.46 ± 0.03	<0.001	0.823

Note. Step time and step length were obtained directly from OptoGait measurements and aver-aged across limbs. Contact time (s) was calculated as the product of gait cycle duration (s) and stance-phase percentage, defined as the sum of loading-response and pre-swing phases. Swing phase represents the complementary percentage of the gait cycle. Cadence conditions corre-spond to 110, 120, and 130 steps·min^−1^. Cadence was experimentally imposed; therefore, the re-ported *p*-value and effect size for cadence are provided solely as a manipulation check and should not be interpreted as inferential results. Partial eta squared (ηp^2^) is reported as a measure of effect size for repeated-measures ANOVA (0.01 = small, 0.06 = medium, ≥0.14 = large).

**Table 2:** Limb-averaged peak plantar pressure (kPa) across cadence conditions (median [Q1–Q3]) with non-parametric analysis and effect sizes.

Plantar Region	110 Steps·min^−1^	120 Steps·min^−1^	130 Steps·min^−1^	Friedman *p*	Kendall’s W
Hallux	3.32 [2.67–5.73]	3.05 [1.92–4.67]	2.89 [1.82–5.35]	<0.001	0.176
Lesser toes	2.24 [1.48–2.75]	1.85 [0.94–2.54]	1.87 [0.87–2.48]	<0.001	0.152
First ray	4.07 [2.63–5.61]	4.23 [2.33–5.33]	3.79 [2.32–5.38]	0.031	0.073
Central rays	6.92 [4.05–8.82]	7.45 [4.58–9.19]	6.98 [3.95–9.01]	0.007	0.106
Fifth ray	2.25 [1.61–3.20]	2.67 [1.63–3.10]	2.25 [1.53–2.93]	0.047	0.060

Note. Values represent limb-averaged peak plantar pressure (PPP, kPa) and are reported as the median [first–third quartile]. Peak plantar pressure was defined as the maximum value recorded within each plantar region across the entire contact phase of the gait cycle (0–100%), obtained from time-normalized pressure data sampled at successive stance instants. Due to non-normal data distribution, Friedman tests were used to assess differences across cadence conditions. Kendall’s W was reported as a measure of effect size (0.10 = small, 0.30 = moderate, ≥0.50 = large). Cadence conditions correspond to 110, 120, and 130 steps·min^−1^.

**Table 3:** Associations between spatiotemporal parameters and plantar pressure outcomes.

Plantar Region	Outcome	Spearman’s ρ (Range)	*p*-Value (FDR)
Fifth ray	Peak	−0.09 to 0.06	>0.05
Fifth ray	PTI	−0.07 to 0.07	>0.05
Lesser toes	Peak	−0.26 to 0.26	>0.05
Lesser toes	PTI	−0.25 to 0.22	>0.05
Hallux	Peak	−0.28 to 0.29	>0.05
Hallux	PTI	−0.24 to 0.35	>0.05
First ray	Peak	−0.15 to 0.16	>0.05
First ray	PTI	−0.27 to 0.27	>0.05
Central rays	Peak	−0.10 to 0.04	>0.05
Central rays	PTI	−0.05 to 0.03	>0.05

Note: Spearman’s rank correlation coefficients (ρ) summarize the range of observed associations between spatiotemporal variables (cadence, step time, contact phase, stride length, step length, and swing phase) and plantar pressure metrics (peak pressure and pressure–time integral, PTI) for each plantar region. After false discovery rate (FDR) correction for multiple comparisons, no correlations remained statistically significant (all p_FDR > 0.05). Detailed correlation coefficients and adjusted *p*-values are provided in Supplementary Table S1.

## Data Availability

The data presented in this study are available upon reasonable request from the corresponding author. The data are not publicly available due to privacy restrictions.

## Supplementary Materials

The following supporting information can be downloaded at: https://www.mdpi.com/article/10.3390/jfmk11010053/s1, Supplementary Table S1: Exploratory correlations between spatiotemporal parameters and regional plantar-pressure metrics.
