# Supplementary material for: Kinematic and Kinetic Adaptations to Step Cadence Modulation During Walking in Healthy Adults
Source: J Funct Morphol Kinesiol. 2026 Jan 26;11(1):53. doi: 10.3390/jfmk11010053 (PMC12922027; doi:10.3390/jfmk11010053)
Supplement: Supplementary file 1 [file jfmk-11-00053-s001.zip › jfmk-4085685-supplementary.pdf]

Supplementary Materials

Supplementary Table S1. Exploratory correlations between spatiotemporal parameters and regional plantar-pressure metrics.

Plantar region: Hallux

p-value (FDR)	Spearman's ρ	Outcome	Spatiotemporal variable
0.421	0.28−	Peak	Cadence
0.483	0.24−	PTI	Cadence
0.688	0.17−	Peak	Contact phase
0.483	0.21−	PTI	Contact phase
0.421	0.29	Peak	Step length
0.421	0.35	PTI	Step length
0.483	0.21	Peak	Step time
0.483	0.21	PTI	Step time
0.421	0.28	Peak	Stride length
0.421	0.34	PTI	Stride length
0.688	0.17	Peak	Swing phase
0.483	0.21	PTI	Swing phase

Plantar region: Lesser toes

p-value (FDR)	Spearman's ρ	Outcome	Spatiotemporal variable
0.483	0.22−	Peak	Cadence
0.440	0.25−	PTI	Cadence
0.421	0.26−	Peak	Contact phase
0.483	0.22−	PTI	Contact phase
0.688	0.15	Peak	Step length
0.849	0.10	PTI	Step length
0.961	0.01−	Peak	Step time
0.909	0.02	PTI	Step time
0.688	0.16	Peak	Stride length
0.849	0.10	PTI	Stride length
0.421	0.26	Peak	Swing phase
0.483	0.22	PTI	Swing phase

Plantar region: First ray

p-value (FDR)	Spearman's ρ	Outcome	Spatiotemporal variable

0.688	0.16	Peak	Cadence
0.695	0.14	PTI	Cadence
0.688	0.14−	Peak	Contact phase
0.421	0.27−	PTI	Contact phase
0.707	0.13−	Peak	Step length
0.713	0.13−	PTI	Step length
0.849	0.10−	Peak	Step time
0.688	0.15−	PTI	Step time
0.688	0.15−	Peak	Stride length
0.688	0.15−	PTI	Stride length
0.688	0.15	Peak	Swing phase
0.421	0.27	PTI	Swing phase

Plantar region: Central rays

**p-value (FDR)**	**Spearman's ρ**	**Outcome**	**Spatiotemporal variable**
0.909	0.04	Peak	Cadence
0.909	0.05−	PTI	Cadence
0.909	0.04	Peak	Contact phase
0.909	0.03−	PTI	Contact phase
0.849	0.10−	Peak	Step length
0.909	0.04−	PTI	Step length
0.909	0.03−	Peak	Step time
0.952	0.01	PTI	Step time
0.864	0.09−	Peak	Stride length
0.909	0.03−	PTI	Stride length
0.909	0.03−	Peak	Swing phase
0.909	0.03	PTI	Swing phase

Plantar region: Fifth ray

**p-value (FDR)**	**Spearman's ρ**	**Outcome**	**Spatiotemporal variable**
0.909	0.02	Peak	Cadence

0.909	0.04	PTI	Cadence
0.909	0.06−	Peak	Contact phase
0.909	0.07−	PTI	Contact phase
0.909	0.08−	Peak	Step length
0.909	0.05−	PTI	Step length
0.909	0.03	Peak	Step time
0.926	0.02−	PTI	Step time
0.864	0.09−	Peak	Stride length
0.909	0.07−	PTI	Stride length
0.909	0.06	Peak	Swing phase
0.909	0.07	PTI	Swing phase

Note. Spearman’s rank correlation coefficients (ρ) were calculated for exploratory regional analyses. None of the regional correlations remained statistically significant after false discovery rate (FDR) correction.
